# Using Rasch Modeling to Re-Evaluate Rapid Malaria Diagnosis Test Analyses 

**DOI:** 10.3390/ijerph110706681

**Published:** 2014-06-26

**Authors:** Dawit G. Ayele, Temesgen Zewotir, Henry Mwambi

**Affiliations:** School of Mathematics, Statistics and Computer Science University of KwaZulu-Natal, Pietermaritzburg, Private Bag X01, Scottsville 3209, South Africa; E-Mails: zewotir@ukzn.ac.za (T.Z.); MwambiH@ukzn.ac.za (H.M.)

**Keywords:** differential item functioning, item response, latent trait, rapid diagnostic test, reliability

## Abstract

The objective of this study was to demonstrate the use of the Rasch model by assessing the appropriateness of the demographic, social-economic and geographic factors in providing a total score in malaria RDT in accordance with the model’s expectations. The baseline malaria indicator survey was conducted in Amhara, Oromiya and Southern Nation Nationalities and People (SNNP) regions of Ethiopia by The Carter Center in 2007. The result shows high reliability and little disordering of thresholds with no evidence of differential item functioning.

## 1. Introduction

The Rasch model is a model used in the design, analysis and scoring of tests, questionnaires and similar instruments, for measuring abilities, attitudes or other variables. Rasch models are a class of probabilistic models that explain the response of a person to a set of items. Item response theory (IRT) concerns models and methods where the responses to variables are assumed to depend upon nonmeasurable respondent characteristics and on item characteristics. The responses to the items (generally binary or polytomous ordinal variables) and the latent trait are linked nonlinearly. As a link function, the logistic function is often used. The Rasch models consider a unidimensional latent trait [1]. The responses to items are influenced by a unidimensional variable characterizing the individuals. To perform Rasch models, general statistical software packages like Stata, R, WINSTEPS or SAS allow estimating parameters in the scope of generalized linear mixed models. In addition to these software packages, RUMM software can also be used. The literature on IRT in general and Rasch models in particular is presented in many research articles and books [1,2,3,4,5,6,7].

Although the Rasch model has been widely used in education, its use in other fields, for example in the health sciences [8], is very limited. The study introduces an item response theory (IRT) model, in particular the Rasch analysis, and shows how it can be applied in the rapid diagnostic tests (RDT) for malaria. This method is important for examining how well the observed data fits the expectations of the measurements provided by the unidimensional results. In other words the Rasch analysis is used to evaluate if the factors set by Ayele, Zewotir and Mwambi in their previous studies ([9,10,11,12]) provide a total score in the malaria RDT up to the model’s expectation onset. Secondly, we examine where the group demonstrates a reliable regular difference in the responses to a factor, through the entire range of the unidimensional latent trait being measured.

## 2. Methods and Materials

In the highland areas of Ethiopia, the incidence of malaria is frequent [13,14] between 1000–2000 meters above sea level [15,16,17]. Therefore, prompt identification and treatment is essential, with microscopy remaining the standard method of diagnosis [18,19], but for most health facilities this method is inaccessible and too expensive. Because of this it is necessary to introduce another method: rapid diagnostic tests (RDT) for malaria. The use of RDTs is important for providing fast treatment based on results and avoiding treatment which is unnecessary. 

In 2007, The Carter Center was responsible for conducting the malaria baseline household cluster survey. To conduct the survey, a questionnaire (the Malaria Indicator Survey (MIS) Household Questionnaire) was used, covering 224 clusters with each cluster containing 25 households. In the survey a listing was made separately for each room and from the information obtained, such as use of mosquito netting, it was possible to ascertain the density of sleepers per room as well as how many sleeping rooms were inside and outside each house. 

To test for the presence of malaria parasite, consent was requested from residents of even-numbered households. A blood sample was collected by taking fingerprick samples from participants for malaria RDT. After the test, if the participants were found to be positive, then treatment was offered and conducted following the national guidelines. Details and extended discussions about the data are available from Ayele, Zewotir and Mwambi [9,10,11,12].

The malaria data was fitted to the Rasch model using the RUMM2030 software. The objective was to examine how well the malaria RDT observed data fitted the expectations of the measurement model. To check the accuracy of the model, three overall Fit statistics can be considered. These methods are person-reliability and item-reliability measures. Person-reliability indicates how likely it is to be able to get the same ordering of individuals for repeated tests. This test is equivalent to the traditional test of reliability. Therefore, high person-reliability indicates that some persons score higher and some score lower, which implies consistency of inferences. On the other hand, item-reliability indicates the ability of the test to define a distinct hierarchy of items along the measured variable on a 0 to 1 scale. The higher number of item-reliability indicates higher reliability of the result.

In addition, using these methods, it can be transformed to approximate a z score. The z score represents standardized normal distribution. Furthermore, if the items and persons are suitable for the model, it is expected to see a mean of approximately zero and a standard deviation of one. The other method is an item-trait interaction statistic. This statistic is reported as a chi-square and reflects the property of invariance across the trait. Therefore, if the chi-square is significant it means the hierarchical ordering of the items varies across the trait and the value compromises the required property of invariance. 

Besides these overall summary fit statistics, individual person and item-fit statistics are presented, both as residuals and as a chi-squared statistic. The residual value between ±2.5 indicates a satisfactory fit to the model. In addition to this, misfit to the model can also be viewed graphically where the observed model fit are groups of respondents across class intervals. The graph can be plotted against the expected model curve (item characteristic curve (ICC)). The ICC is the expected value curve which takes a logistic functional form. Items with good fit will show each of the group plots lying on the curve. However, plots which are steeper than the curve would be considered as over-discriminating and those flatter than the curve considered under-discriminating. The summary of overall chi-square for items is given as the item trait interaction statistic. In the analysis, Bonferroni corrections are applied to adjust the chi-squared *p*-value [20]. This is done to account for multiple testing.

Furthermore, examination of person fit is important for item fit. If there are few respondents who deviate from model expectation, this may cause significant misfit at the item level. In case of validation of a scale, the misfit runs the risk of discarding the scale. But the scale would be more appropriate to find out why a few respondents may be responding in a way different to others. An indication of how well-targeted the items are in the sample can be obtained from a comparison of the mean location score obtained for the persons with that of the value of zero set for the items. For a well-targeted measure the mean location would also be around the value of zero. A positive mean value indicates that the sample as a whole was located at a higher level than the average of the scale. On the other hand, a negative value would suggest the opposite. 

From the analysis, the internal consistency reliability estimate of the scale can be obtained. This is obtained based on the Person Separation Index (PSI). To calculate the reliability, the logit scale estimates for each person are used. To see the improvement of scale construction, the sources of deviation from model expectation can be examined. A good fitting model can be obtained for each of the items. This is achieved for high level attribute measurement if the measurement would indorse high scoring responses. But low scoring endorsement is obtained for individuals with low levels of the attribute. In Rasch analysis, thresholds can be used. The use of thresholds is to indicate an ordered set of responses for each of the items. The term threshold refers to the point between two response categories, *i.e.*, either response is equally probable. 

To investigate responses to an item, the category probability curves can be inspected. For a well-fitting item, it can be expected across the whole range of the trait to be measured. In addition to this, each response option would systematically take turns showing the highest probability of endorsement. Disordered thresholds indicate the most common source of item-misfit, *i.e.*, the failure of respondents to use the response categories in a manner consistent with the level of the trait being measured. Disordered thresholds occur for respondents with difficulty consistently discriminating between response options. The problem can occur for too many response options and when the labelling of options is potentially confusing. To overcome this problem, collapsing of categories where disordered thresholds occur, improves overall fit to the model. 

Differential item functioning (DIF) is the other issue that can affect model fit in the form of item bias. This occurs when different groups within the sample respond in a different manner to an individual item. This can occur despite equal levels of the underlying characteristic being measured. From the analysis, two types of DIF may be identified. DIF’s also show a consistent systematic difference in their responses to an item. This is referred to as uniform DIF. When there is non-uniformity in the differences between the groups then this is referred to as non-uniform DIF. When non-uniformity is detected, the problem can be remedied by splitting the file by group and separately calibrating the item for each group. But, there is little that can be done to correct the problem. Therefore, it is often necessary to remove the item from the scale. 

In RUMM, the statistical and graphical methods can be used to detect the presence of DIF. Analysis of variance is conducted for each item comparing scores across each level of the person factor and across different levels of trait. Uniform DIF is indicated by a significant main effect for the person factor, and the presence of non-uniform DIF is indicated by a significant interaction effect. 

A principal component analysis (PCA) of residuals can be used to detect the sign of multidimensionality when there are issues of threshold disordering and DIF. If there is no meaningful pattern of residuals, the result suggests the assumption of local independence. This leads to unidimensionality of the scales. Moreover, the subsets of items can be determined by allowing the factor loading of the first residual. The use of a paired t-test helps to see if the person estimate derived from the subsets significantly differs from that derived from all items. Furthermore, violation of the assumption of local independence can be detected if the person estimate is found to differ between the subset and the full scale [2,21,22].

## 3. Results and Discussion

For the RUMM analysis, baseline household cluster malaria survey which was conducted by The Carter Center in 2007 was used. Malaria RDT results, indoor residual spraying and use of mosquito nets were used as person items. The other variables considered as items were: main source of drinking water; time to collect water; toilet facilities; availability of electricity radio and television; total number of rooms; main material of the room’s walls, roof and floor; total number of nets; region; altitude; age and family size. For the analysis, altitude, age, total number of rooms and family size were categorized to be appropriate for the RUMM2030 analysis.

The residual mean value for items in the anxiety subscale is 0.0205 with a standard deviation (SD) of 1.0187. To be a good fit, SD is expected to be close to 1 and since the value is close to 1, the fit is an adequate fit to the model. This result is supported by a non-significant chi-squared interaction of 96.994 with *p*-value = 0.3491. Therefore, the scale fits the Rasch model. The value of the Person-Separation-Index for the original set of sixteen items with the response categories was 0.832. This result indicates that the scale worked well to separate the persons. The Power of Test-of-Fit is a visual representation of the Person-Separation-Index. It is indicative of the power of the construction to discriminate amongst the respondents. Based on the values, 0.7 is the minimum accepted level of Person-Separation-Index. This value indicates that it is possible to differentiate statistically between two groups of respondents. Furthermore, a value of 0.9 means that we can statistically differentiate between four or more groups. The Person-Separation-Index is also an indicator of how much we can rely on the Fit Statistics. If the Person-Separation-Index is low, then the Fit Statistics that have been obtained may not be reliable as there will be a substantial amount of error surrounding them. If the Person-Separation-Index is high, then the Fit Statistics that have been generated can be deemed to be more reliable. Based on this, because our Person-Separation-Index is 0.832, it can be concluded that the Fit Statistics are reliable.

**Figure 1:**
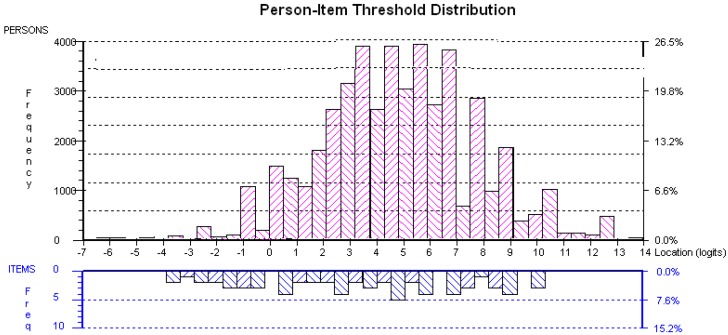
Person-item threshold distribution (16 items).

Figure 1 shows the location of the item threshold estimates relative to the distribution of person-item set consisting of all items for the original data. From the figure it can be seen that the person locations are positively skewed. To find person-item threshold distribution, person and item locations are logarithmically transformed and plotted on the same continuum. The plot common unit of measurement was termed as logit. The ordinal data was converted as equal-interval data. Furthermore, Figure 1 illustrates how person and item locations can be plotted on the same continuum along the *x* axis. The upper part of the graph represents groups of respondents who have tested for malaria infection and their ability to respond to the questions. The lower part of the graph represents the item locations and their distribution. Both respondent’s ability level and item difficulty level are being shown on the same linear scale. Some items are located in the same place in terms of difficulty and this common location is represented as one block on top of another. A lot of item thresholds are clustered around the central locations. From the plot, endpoints are known as the floor and ceiling of the scale. The respondents that are located outside of the range measured by the scale were not included in the analysis but excluded as extreme scores. 

It can be seen that little information was derived from those respondents with maximum scores (at the top end of the scale). Maximum information for any given item is derived when the respondents have the same logit ability as the item’s logit difficulty. Besides the person-item threshold distribution, another useful function of this display screen is the option to look at the location, or ‘ability’ differences between person factor RDT result, use of anti-malaria spray and use of mosquito net groups. Moreover, the statistical relationship between person factors (RDT result, use of anti-malaria spray and use of mosquito nets) can be assessed. To test the assumption whether there is a statistical difference between the person factor groups, *i.e.*, malaria RDT, use of mosquito nets and use of indoor residual spraying, the ANOVA can be used. The ANOVA value is given in Table 1. The result from the ANOVA analysis reveals that there is a statistical difference in ability between malaria RDT result of positive and negative subgroups (*p* = 0.00156). Similarly, there is a statistical difference between use of indoor residual spraying and not using (*p* = 0.00327), and between respondents who are using and not using mosquito nets (*p* = 0.006027). 

**Table 1:** ANOVA table for malaria RDT results, indoor residual spraying and use of mosquito nets.

Source	Sum of squares	DF	Mean sum of squares	F-Stat	Prob
Malaria RDT result					
Between	6.28	1	6.28	14.81	0.00156
Within	6,408.78	15,119	0.42		
Total	6,415.06	15,120			
Indoor residual spraying					
Between	87.75	1	87.75	209.68	0.00327
Within	6,327.31	15,119	0.42		
Total	6,415.06	15,120			
Use of mosquito nets					
Between	68.47	1	68.47	163.02	0.006027
Within	6,346.59	15,119	0.42		
Total	6,415.06	15,120			

DF = degree of freedom.

Targeting and reliability is used to measure the suitability to the analysis. The other inspection method is the graphical inspection of the ICC for each item. Using ICC, the fit of expected and observed values was examined. Using the ICC graphical method, it is possible to represent the average response of persons within each class interval (CI) and expected values. Alternately, DIF can be used to diagnosis the model.

For DIF analysis, there are two groups and we consider the two groups as being of equal status. In the use of DIF the perspective is that there is a standard or main group, and there is a subgroup sometimes referred to as a focal group, which might have items which are biased. When using DIF analysis, the sample sizes of the two groups should be as close as possible. This is because if the sample sizes are different and there is DIF, then the estimates will be weighted by the estimates that would be present for a group with a larger sample size. 

The use of analysis of variance for residuals provides the capability to identify two kinds of DIFs. These are uniform DIF and non-uniform DIF. The two-way ANOVA structure involves the class intervals as one of the factors, and the groups as the other factor. Then it is possible to study the main effect of the class intervals, the main effect of the groups and the interaction between the two. The main effect across class intervals is a general test of fit of the responses to the ICC, irrespective of any classification by groups. Items can show fit to the model using this criterion, while showing DIF. 

Non-uniform DIF occurs when the observed means of responses in the class intervals of two groups are different systematically. In ANOVA, there is an interaction between the class intervals and the groups. If there is no non-uniform DIF, then uniform DIF can be interpreted directly. Uniform DIF occurs where the observations of responses in the class intervals of two groups are different systematically and are parallel. This means that for the best estimate of locations of persons on the continuum one group tends to have a higher mean than the other group. 

Groups can have different means, but some items have DIF. This means that DIF detects an interaction between some items and the rest of the items, not an absolute effect. Suppose an item has DIF. Then suppose a whole set of items that has this characteristic are put together, and they all individually show DIF in the same direction. Then these items put together would show no DIF, but the mean of one group would be greater than the other.

The initial summary of DIF for malaria RDT result, use of indoor residual spraying and use of mosquito nets shows misfit across the continuum as evidenced by the Class Interval for malaria RDT result, indoor residual spraying and use of mosquito nets. These items are item 5 (total number of rooms) due to malaria RDT, items 5, 6, 7 and 11 (total number of rooms, total number of nets, sex and wall material) due to indoor residual spraying and items 1 and 5 (region and total number of rooms) to use of mosquito nets. To resolve this problem, there are suggestions for the correction of the significance level in the literature, and a common one is the Bonferroni correction. This is very simple to carry out; the chosen probability value of significance is simply divided by the number of tests of fit. There is some controversy with this correction. In RUMM, both the numbers with correction and the numbers without correction are provided to give the user discretion in making decisions. It also permits them to report both. Table 2 shows the ANOVA of residuals after misfitted items have been resolved for malaria RDT result. Therefore no item shows any misfit. Similarly, for indoor residual spraying and use of mosquito nets the residuals were assessed to identify if there is misfit to the model. From the result it was found that there was no item misfit.

Diagnosis and detection of violations of independence can be reflected in the fit of data to the model. Over-discriminating items often indicate response dependence. This situation indicates multidimensionality. Response dependence increases the similarity of the responses of persons across items. Therefore, responses are more Guttman-like than they should be under no dependence. Multidimensionality acts as an extra source of variation in the data, and the responses are less Guttman-like than they would be under no dependence. Violations of local independence can be assessed by examining patterns among the standardized item residuals. High correlations between standardized item residuals indicate a violation of the assumption of independence. A principal component analysis (PCA) of the item residuals provides further information about dependence. After extracting the ‘Rasch factor’ there should be no further pattern among the residuals. If a PCA indicates a meaningful pattern the scale or test is not unidimensional. 

**Table 2:** DIF Summary with Bonferroni corrected for malaria RDT result.

Item	Class Interval		RDT		Interaction	
	*F*	*p*-value	*F*	*p* -value	*F*	*p* -value
Region	15.182	0.082	4.678	0.290	2.884	0.491
Availability of electricity	41.121	0.074	3.749	0.315	2.918	0.516
Availability of radio	2.534	0.239	1.967	0.089	2.952	0.541
Availability of television	4.951	0.111	4.703	0.043	2.986	0.567
Total number of rooms	3.826	0.214	3.968	0.050	3.054	0.617
Number of nets	1.660	0.240	3.996	0.096	3.088	0.642
Gender	4.724	0.265	4.023	0.143	3.122	0.667
Source of drinking water	4.387	0.290	4.050	0.189	3.157	0.692
Distance to get water	2.686	0.315	4.077	0.235	3.191	0.717
Toilet facility	5.329	0.340	4.104	0.282	3.225	0.743
Wall material	4.746	0.365	4.132	0.328	3.259	0.768
Roof material	1.220	0.390	4.159	0.374	3.293	0.793
Floor material	3.700	0.416	4.186	0.421	3.327	0.818
Family size	5.294	0.441	4.213	0.467	3.361	0.843
Age group	2.685	0.466	4.240	0.513	3.395	0.868

The results of a PCA on a data set with items sorted according to their loadings on principal component one (PC1) shows no meaningful pattern. Therefore, the scale or test is unidimentional. Table 3 shows the summary of the PCA. The eigenvalue of 2.42 for the first component is considerably larger than the eigenvalues for the other components. The first principal component explained 15.14% of the total variance among residuals. This all suggests unidimensionality with items 1 to 16 tapping into a second factor, after the main factor had been extracted. 

**Table 3:** Summary of the PCA.

PC	Eigen	Percent	CPercent	StdErr
Region	2.422	15.14%	15.14%	0.332
Electricity	1.642	10.26%	25.40%	0.221
Radio	1.539	9.62%	35.02%	0.204
Television	1.288	8.05%	43.06%	0.169
Total Number of Rooms	1.204	7.53%	50.59%	0.158
Number of nets	1.105	6.91%	57.50%	0.143
Sex	1.05	6.57%	64.06%	0.137
Source of drinking water	0.946	5.91%	69.97%	0.121
Distance to get water	0.879	5.49%	75.46%	0.108
Toilet facility	0.817	5.10%	80.57%	0.107
Wall material	0.719	4.50%	85.07%	0.098
Roof material	0.677	4.23%	89.29%	0.093
Floor material	0.622	3.89%	93.18%	0.086
Family size	0.566	3.54%	96.72%	0.08
Age group	0.483	3.02%	99.74%	0.078
Altitude	0.041	0.26%	100.00%	0.054

## 4. Conclusions

The findings of this study suggest that the Rasch model can be utilized for measuring the level of malaria risk as a single latent concept and for establishing the relative degree of severity of each type of socio-economic, demographic and geographic factors. The measurement scale provides sources of reference to develop a scale that is normed on the malaria situation in Ethiopia. Furthermore, the result provides a useful measurement tool to indicate, design and assess the malaria problem focusing on socio-economic, demographic and geographic factors. 

The initial descriptive analysis of the frequency distributions shows that the sixteen items scale with each response categories mistargeted the current sample. This was indicated by plenty of very few responses in the categories representing low self-efficacy. This conclusion was confirmed and the analysis elaborated taking advantage of the Rasch model that places independently estimated item and person parameters. 

The Rasch analysis supports the measurement properties, internal consistency reliability, targeting, and unidimensionality of the different levels of malaria RDT result, use of indoor residual spraying and use of mosquito nets. During the analysis, it was necessary to remove some items from each of the scales to achieve fit to the Rasch model. Using differential item functioning analysis, it was found for malaria RDT result, use of indoor residual spraying and use of mosquito nets the items responding well. The categorization of the items was examined using the Rasch model for the ordering of the item thresholds. From the analysis, few items showed disordered thresholds indicating some problems with the categorization of items. 

In conclusion, application of the Rasch model in this study has supported the viability of a total of sixteen items for measuring malaria RDT results, use of indoor residual spraying and use of mosquito nets. Therefore, from the analysis it can be seen that the scale shows high reliability. But, there were little disordering of thresholds and no evidence of differential item functioning. In general, the Rasch model for malaria RDT observed data, reasonably met the unidimensionality and local independence assumptions. Furthermore, high consistent item reliability indices, acceptable item difficulty invariance and infit and outfit values were obtained. The malaria study evaluates a wide range of socio-economic, demographic and geographic factors. The scale of socio-economic, demographic and geographic factors is appropriate for the use to measure the level of malaria RDT results in Ethiopia. Because the measurement scale is at the interval level, it provides a useful measurement tool to inform, design and evaluate interventions that target use of socio-economic, demographic and geographic factors.
